# Looking forward to new targeted treatments for chronic spontaneous urticaria

**DOI:** 10.1186/s13601-016-0139-2

**Published:** 2017-01-10

**Authors:** Emek Kocatürk, Marcus Maurer, Martin Metz, Clive Grattan

**Affiliations:** 1 Department of Dermatology, Okmeydanı Training and Research Hospital, Istanbul, Turkey; 2 Department of Dermatology and Allergy, Charité – Universitäts medizin, Berlin, Germany; 3 St John’s Institute of Dermatology, Guy’s Hospital, London, UK

**Keywords:** Chronic spontaneous urticaria, New treatment targets, Pathogenesis, Targeted treatment, Future treatments

## Abstract

The introduction of omalizumab to the management of chronic spontaneous urticaria (CSU) has markedly improved the therapeutic possibilities for both, patients and physicians dealing with this disabling disease. But there is still a hard core of patients who do not tolerate or benefit from existing therapies and who require effective treatment. Novel approaches include the use of currently available drugs off-licence, investigational drugs currently undergoing clinical trials and exploring the potential for therapies directed at pathophysiological targets in CSU. Off-licence uses of currently available drugs include rituximab and tumour necrosis factor inhibitors. Ligelizumab (anti-IgE), canakinumab (anti-IL-1), AZD1981 (a PGD2 receptor antagonist) and GSK 2646264 (a selective Syk inhibitor) are currently in clinical trials for CSU. Examples of drugs that could target potential pathophysiological targets in CSU include substance P antagonists, designed ankyrin repeat proteins, C5a/C5a receptor inhibitors, anti-IL-4, anti-IL-5 and anti-IL-13 and drugs that target inhibitory mast cell receptors. Other mediators and receptors of likely pathogenic relevance should be explored in skin profiling and functional proof of concept studies. The exploration of novel therapeutic targets for their role and relevance in CSU should help to achieve a better understanding of its etiopathogenesis.

## Background

Chronic spontaneous urticaria (CSU) is defined as the spontaneous appearance of itchy weals, angioedema, or both, for at least 6 weeks [1]. It is a self limiting disorder, persisting for 2–5 years in the majority of cases, although 20% of patients suffer for more than 5 years [2]. Beyond the visual impact of weals and angioedema, quality of life is substantially reduced in patients due to interference with sleep, daily activities, social interaction, work productivity [3] and emotional well-being [4]. There is also a high socioeconomic impact from both the direct (medication and healthcare visits) and indirect costs (absence from or reduced efficiency while at work) [5, 6].

The introduction of omalizumab as an add-on therapy to H1 antihistamines as a management option has markedly improved the therapeutic possibilities for both CSU patients and physicians dealing with this chronic disease. Nevertheless, there are still many patients who do not tolerate or benefit from existing therapies including omalizumab.

This review describes possible future treatment options and novel therapeutic targets in CSU based on the pathophysiology of the disease and summarizes ongoing clinical studies in CSU.

## Pathophysiological events in chronic spontaneous urticaria

Understanding the pathophysiology of urticaria is important for the identification of potential targets for novel treatments. Weals and angioedema in CSU result from the degranulation of skin mast cells, which release histamine, proteases and cytokines with generation of platelet-activating factor and other arachidonic acid metabolites (prostaglandin D2, leukotrienes C4, D4 and E4). These mediators induce vasodilatation, increase vascular permeability, and stimulate sensory nerve endings that lead to swelling, redness and itch [7].

The weal is characterized by dermal oedema, vasodilatation and a perivascular mixed infiltrate composed of predominantly CD4+ lymphocytes with variable numbers of monocytes, neutrophils, eosinophils and basophils similar to allergen-mediated late-phase skin reactions, but the cytokine profile is characterized by an increase in IL-4, IL-5 and interferon-gamma, which is suggestive of a mixed Th1/Th2 response [8–10]. Cytokines that promote a Th2 profile of inflammation [IL-33, IL-25 and thymic stromal lymphopoietin (TSLP)], are increased in lesional but not uninvolved skin [11]. Vascular markers, with eosinophil and neutrophil infiltration, are dominant in lesional skin, whereas eosinophils and microvascular changes persist at uninvolved sites by comparison with healthy controls. They may prime the skin for further wealing in conjunction with increased mast cell numbers [12]. Biopsies from both lesional and nonlesional skin of CSU patients show upregulation of soluble mediators and adhesion molecules, which is indicative of a “widespread immunologic activation” possibly lowering the threshold of mast cell degranulation to triggering stimuli [13–15]. Some authors suggest that CSU is an “immune-mediated inflammatory disorder” that involves an immunological activation event following exposure to an exogeneous or modified endogeneous trigger (such as functional autoantibodies) in the presence of susceptibility factors (e.g. stress, pathogen exposures) [16]. The inflammatory cascade in CSU may be modulated by an altered chemokine–cytokine network and is attributed to immune dysregulation as a consequence of disturbed innate and adaptive immunity [15].

The mechanisms by which cutaneous mast cells are activated to induce hives in CSU are still not completely understood. It is widely accepted that CSU is due to autoimmune/autoreactive mechanisms in some patients. There is considerable evidence for a role and clinical relevance of functional IgG autoantibodies to IgE or to the extracellular α subunit of the high affinity IgE receptor (FcεRIα) in approximately 30–50% of patients [17, 18]. These autoantibodies belong mainly to the complement fixing and activating subtypes IgG1 and IgG3. The activation of complement generates C5a, which interacts with the C5a receptor on the surface of skin mast cells and induces activation [19].

The stimulus for mast cell activation in the remaining 50–70% of urticaria patients is less clear. They potentially include IgE antibodies against autoallergens, neuropeptides such as substance P, alarmins and complement activation due to chronic infections [20, 21]. The relevance of observed coagulation abnormalities on mast cell degranulation is uncertain although a role for thrombin generated by extrinsic factor activation has been proposed [22].

Basophils also appear to be involved in the pathogenesis of CSU. Peripheral blood basopenia is seen in patients with high disease activity and may be explained by the recruitment of basophils from the blood into skinlesions [23]. CSU basophils also show functional abnormalities during active disease that revert during disease remision. Greaves was first to show the hyporesponsiveness of basophils of patients with CSU to anti-IgE [24]. Reduced basophil responsiveness to anti-IgE and altered signal transduction are reportedly seen in at least half of the patients [24–28]. Vonakis and coworkers reported basophil hypo-responsiveness in about half of the patients with chronic urticaria that is linked to excessive activity of the negative regulator Src homology inositol phosphatase (SHIP). SHIP dephosphorylates kinases such as spleen tyrosine kinase (Syk) and consequently decreases cell responsiveness. Reversal of anti-IgE hypo-responsiveness was observed upon disease remission, which suggests a relationship with the disease pathogenesis [28].

The current guidelines for CSU recommend the use of non-sedating H1 antihistamines followed by leukotriene antagonists (LTRA), ciclosporin and omalizumab as add-on treatment to antihistamines. Although histamine is a major contributor, approximately 40–55% of patients are refractory and achieve little or no benefit even from updosing antihistamines [29]. Leukotriene inhibitors are not superior to placebo or H1 antihistamines and should be used in combination with an antihistamine [30]. Ciclosporin is often effective, especially in patients with a positive basophil histamine release assay [31], but some patients do not tolerate treatment or have to be discontinued due to adverse events. Ciclosporin is generally used in short courses but long term treatment with low doses has been reported to be safe and effective [32]. Omalizumab has provided a substantial advance in the treatment of CSU, but not everyone responds, the drug is expensive and is not readily available for many patients in many countries.

## Off-label use of licensed drugs

### Anti-TNF therapies

TNF-α antagonists have been reported to be effective in 60% of 20 CSU patients of a retrospective case series [33], including some omalizumab non-responders, and TNF-α has been reported to be upregulated in patients with CSU as compared to healthy controls [34].

### Anti-CD20

Rituximab, a chimeric monoclonal anti-CD20 has been successfully used for the treatment of several autoimmune diseases [35]. Treatment of CSU patients with rituximab hasbeen reported; it was effective in two case reports with autoimmune urticaria [36, 37], but was ineffective in another [38]. There has been a Phase I/II study of rituximab in patients with CSU, which was registered in clinicaltrials.gov (NCT00216762). This study was halted by FDA due to safety concerns. Rituximab could be an option in very resistant autoimmune urticaria cases, but the side effect profile of this drug needs to be taken into consideration.

## Drugs that are under investigation

### Anti-IgE therapies

Even though omalizumab is highly effective in CSU [39–41] and can reduce disease activity in many forms of inducible urticarias [42, 43], there are patients who do not benefit sufficiently or at all from it. Strategies for these patients are evolving and may include off-label combinations of omalizumab with current immunosuppressive or anti-inflammatory drugs. MEDI-4212, ligelizumab (QGE031) and quilizumab are new anti-IgE reagents that are currently undergoing phase 2 trials testings [44]. Quilizumab and ligelizumab are under investigation in CSU.

#### Ligelizumab (QGE031)

Ligelizumab is a humanized IgG1 monoclonal antibody that binds with high affinity to the Cε3 domain of IgE. Compared to omalizumab, ligelizumab shows six-fold to nine-fold greater suppression of allergen-induced skin prick tests in vivo. The estimated plasma half-life is 20 days with over 95% suppression of allergen-induced skin prick test responses 6 weeks post dose by comparison with 41% for omalizumab. It also provides greater and longer suppression of free IgE and IgE on the surface of circulating basophils as compared to omalizumab [45]. These findings suggest that ligelizumab may be more potent than omalizumab in the treatment of CSU. There is an ongoing multi-center, randomized, double-blind, placebo, and active-controlled phase 2b dose-finding study of ligelizumab as add-on therapy to investigate the efficacy and safety in patients with CSU (NCT02477332). The effects of ligelizumab and omalizumab as control, in this trial, are assessed by measurements of wheal numbers, itch intensity and the urticaria activity scores at baseline, at week 12 and at week 20. The study includes four different doses of ligelizumab given as subcutaneous injections of omalizumab 300 mg monthly as a positive control and a placebo arm. The estimated enrolment is 360 CSU patients and anticipated study completion date is March 2017.

#### Quilizumab

Quilizumab is a humanized monoclonal antibody that targets the M1 prime segment of membrane expressed IgE on IgE-switched B cells and plasmablasts. Quilizumab is in clinical development for the treatment of allergic asthma. By causing the depletion of IgE-switched B cells and plasmablasts, it reduces serum IgE levels [46]. These findings suggest that quilizumab may be an effective treatment of CSU. A recently performed multicenter, double-blind study with 32 adult CSU patients who were symptomatic despite H1 antihistamine treatment with or without LTRAs looked at responses to placebo (n = 17) or 450 mg quilizumab (n = 15) given subcutaneously at Day 1 and Day 29 [47]. The absolute change from baseline to week 20 in the weekly itch score (the primary efficacy outcome) decreased by 3.3 points and 1.5 points in patients treated with quilizumab relative to the placebo group at week 4 and 20, respectively. These decreases were not significantly different as compared to placebo and did not fall within the minimally important difference of 4.5–5. The reasons for this are likely to include the effects of quilizumab treatment on IgE levels. Quilizumab reduced median serum total IgE only by approximately 30% from baseline at week 20, when it reached its lowest median level. Longer use of quilizumab in CSU patients or its combination with omalizumab may improve treatment effects and lead to sustained responses. This should be explored in future studies.

### Anti-IL-1 therapies

IL-1 is a key inflammatory cytokine of innate immunity. IL-1α and IL-1β both mediate their biologic responses via activation of the IL-1 receptor type I, whereas IL-1Rα functions as a receptor antagonist [48]. In the IL-1β-mediated autoinflammatory diseases Cryopyrin Associated Periodic Syndrome (CAPS) and Schnitzler Syndrome (SchS), non-itchy urticarial rash is a hallmark symptom. Recent findings suggest that IL-1β not only induces urticarial rashes in autoinflammatory diseases, but also plays a role in other allergy-related diseases such as bronchial asthma, contact hypersensitivity and atopic dermatitis [49]. In addition to the dramatic improvement of urticarial rashes in autoinflammatory syndromes upon IL-1 blocking treatments, IL-1 blocking therapies can also be effective in different types of urticaria including delayed pressure urticaria and cold urticaria [50, 51].

Also, the efficacy of anti-IL-1β monoclonal antibody canakinumab is now being evaluated in a phase II randomized double-blind placebo controlled single center study in patients with moderate to severe chronic idiopathic urticaria (URTICANA) (NCT01635127). The pilot study is estimated to enroll 20 patients and the efficacy of subcutaneous injection of 150 mg of canakinumab will be evaluated with urticaria activity scores and daily weal scores 1, 2, 4 and 8 weeks after injection. There is another study evaluating the safety and efficacy of anti-IL-1β receptor blocker rilonacept on cold contact urticaria, which is designed as a double-blind placebo-controlled phase II study (NCT02171416). The primary outcome measure critical temperature threshold will be evaluated at day 42. Secondary outcomes include quality of life assessments and differences in mast cell mediator release. The estimated number of patients to be enrolled is 20 and the anticipated date of completion is April 2017.

### PGD2 receptor antagonists

Prostaglandin D2 (PGD2) is a major product of the COX pathway and has long been implicated in diseases such as asthma and allergic rhinitis. The major source of PGD2 in allergic disease is thought to be the mast cell [52]. PGD2 has three receptors: D-type prostanoid receptor (DP) 1, Chemoattractant Receptor Homologous Molecule expressed on Th2 cells (CRTH2) and the thromboxane receptor, which are expressed by endothelial and airway smooth muscle cells, as well as eosinophils, Th2 cells and basophils. [53–55]. The DP2 receptor CRTH2 is held to be responsible for the pro-inflammatory activities of PGD2in the pathogenesis of asthma and rhinitis [52]. The DP2 antagonist OC000459 has shown promising clinical effects in phase 2 studies in patients with rhino conjunctivitis, asthma and eosinophilic oesophagitis [56–58]. There is an additional CRTH2 antagonist, QAW039, or fevipiprant, that is extensively tested in asthma, but also in allergic rhinitis and AD [59]. Expression of CRTH2 was found to be increased on eosinophils of chronic urticaria patients but not acute urticaria [60]. Sterba et al. suggested that the DP2/CRTH2 pathway may be involved in the recruitment of eosinophils and basophils to CSU skin lesions [61]. There is an ongoing study with AZD1981; an oral, potent, selective, reversible antagonist of CRTH2, which is designed as a phase IIa, randomized, placebo-controlled, double-blinded study to assess its efficacy and safety in patients with CSU who are refractory to H1 antihistamines (NCT02031679). AZD1981, in this trial, is taken at 40 mg twice daily. Primary outcomes measures are urticaria activity scores and secondary outcome measures will be the quality of life benefit provided by treatment and the ability of AZD1981 to inhibit PGD2-induced eosinophil shape which will be assessed on days 21–28 and evaluation of adverse events on 8th week. The study estimates to enroll 48 patients.

### Molecules that target intracellular pathways of mast cell activation following FceRI activation

There is a heightened releasability of mediators from mast cells and basophils in patients with urticaria [62]. Spleen tyrosine kinase (Syk) is a central regulator in promoting histamine release and cytokine, leukotriene and prostaglandin synthesis while Src homology 2 containing inositol phosphatases (SHIP-1 and SHIP-2) have inhibitory activity [63]. Vonakis found a deficiency in SHIP in basophils of CSU patients and Saini showed that in patients with positive histamine release from mast cells upon anti-IgE stimulation, SHIP-2 was lowered and Syk was elevated [64, 65]. A potent and selective Syk inhibitor GSK2646264 is currently being evaluated in a topical formulation in a randomised, double blinded study to assess its safety, tolerability, pharmacodynamics and pharmacokinetics in healthy controls and patients with cold urticaria or CSU. It is formulated as a 0.5 and 1% topical cream (NCT02424799). It Inhibits histamine release in vitro from basophils and mast cell lines, has excellent solubility with good skin penetration, is photostable and has desirable pharmacokinetics for skin delivery with a plasma t1/2, of 57 h so has potential for management of mast cell activation disorders in the skin. The assessments will include tolerability measured with skin irritation scoring system and clinical laboratory safety tests. The study will include 80 patients and the estimated time for completion is November 2016.

A SHIP-1 activator (AQX-1125) is currently under investigation in a clinical study for patients with atopic dermatitis (NCT02324972) and may potentially be another targeted therapy for urticaria in the near future.

A list of drugs under investigation in clinical trials is given in Table 1.

**Table 1:** Drugs under investigation for CSU

Study drug	Type of the drug	Clinicaltrials.gov identifier	Status
Quilizumab	Anti-IgE	NCT01987947	Completed
Ligelizumab	Anti-IgE	NCT02477332	Recruitment closed
AZD1981	PGD2 receptor antagonists	NCT02031679	Data processing
GSK2646264	Syk inhibitor	NCT02424799	Recruiting
Canakinumab	Anti-IL-1	NCT01635127	Unknown
Rilonacept	Anti-IL-1	NCT02171416	Recruiting

*Syk* spleen tyrosine kinase, *PGD2* prostagladin D2, *IL*-*1* ınterleukin-1

## Potential therapeutic targets for novel drug approaches

### Therapies that target neuropeptide-induced inflammation

Neuropeptides canact as mediators of inflammation and are found to be elevated in various allergic diseases including bronchial asthma and atopic dermatitis [66]. A role for neuropeptides in urticaria has been suggested by the observation that substance P (SP) and other neuropeptides induce itch and weal formation in the skin, and SP has been shown to be a mast cell degranulator in vitro [67, 68]. Neuropeptide levels in CSU patients have been investigated in several studies. For example, Metz et al. found that serum SP is upregulated in CSU, and levels were correlated with disease activity [58, 69]. Serum levels of neuropeptides such as neuropeptide Y, vasoactive intestinal peptide, stem cell factor and nerve growth factor were shown to be significantly decreased after treatment with H1 antihistamines in CSU patients [70, 71].

SP binds with different affinities to three neurokinin receptors (NKR 1–3), but mainly to NKR1, which is expressed in the central nervous system and the skin [72]. In recent case reports and case series, SP antagonists demonstrated a significant antipruritic effect in acute and chronic pruritus such as drug-induced pruritus, paraneoplastic pruritus, prurigo nodularis, cutaneous T cell lymphoma, and brachioradial pruritus [73]. No side effects or only mild effects were reported in these studies. Wallengren et al. showed that a SP antagonist, spantide, was able to inhibit immediate and delayed type cutaneous hypersensitivity reactions [74]. The topical application of aprepitant was not found to be successful in 13 patients with pruritic skin conditions [75]. VLY-686 (tradipitant), a novel oral NK-1 receptor antagonist, was investigated in two studies, which are completed but not yet reported: A phase I study (NCT01919944) to investigate its effects in the prevention and reduction of itch and skin reactions induced by SP injections, and a phase II proof of concept study (NCT02004041) to evaluate its efficacy in treatment-resistant pruritus in atopic dermatitis. After this proof of concept study, tradipitant is being investigated in the NCT02651714 study which is recruiting atopic dermatitis patients with treatment resistant pruritus. Currently, aprepitant, serlopitant, tradipitant and orvepitant are under investigation. Studies on the effects of SP antagonists in CSU are needed.

### Therapies that target the IgE–FcεRI interaction

DARPins (designed ankyrin repeat protein) are genetically engineered antibody mimetic proteins that exhibit highly specific and high affinity target protein binding. In contrast to monoclonal antibodies, DARPins are small oligonucleotides that act rapidly, can be used as oral drugs, and are inexpensive to produce. Recently, an IgE-specific DARPin has been reported to bind IgE with very high affinity, causing IgE molecules to dissociate from high-affinity IgE receptors, and suppress mast cell activation [76]. DARPins are promising candidates for the treatment of allergic diseases as well as CSU.

### Therapies that target complement C5a/C5a receptor

Some of the mast cell-activating autoantibodies involved in the pathogenesis of CSU belong to the complement fixing and activating subtypes IgG1 and IgG3 [77]. The binding of these antibodies to FcεRI or IgE leads to the generation of C5a, which interacts with the C5a receptor localized on the surface of MCTC type mast cells (the dominant mast cells of the skin), thereby activating them to release mediators [17]. Fiebiger et al. showed that C5a receptor blockade of basophils or complement depletion substantially reduced the histamine-releasing function of autoantibody-positive sera from CSU patients in vitro [77]. The proinflammatory effects of C5a have been implicated in various pathological conditions making C5a and its receptors attractive targets for therapeutic intervention for inflammatory diseases.

C5a binds to two receptors, C5aR and C5L2, of which C5aR is held to be more important for the proinflammatory effects of C5a. In recent years, potent antagonists for C5aR have been developed including nonpeptide small molecules, C5a mutants, short peptides and cyclic peptides, mAbs and antibody fragments [78]. Eculizumab is a recombinant humanized monoclonal antibody directed against C5. Eculizumab is effective in treating paroxysmal nocturnal hemoglobinuria and atypical haemolytic-uraemic syndrome. It has been suggested that eculizumab may attenuate allergen-induced asthma responses in humans, but the clinical benefit with eculizumab for reducing allergic asthma consequences in humans remains unclear [79]. Eculizumab and other C5a antagonists currently under development should be assessed for their efficacy and safety in CSU.

### Therapies that target the IL-5/eosinophil pathway

There are a number of reports emphasizing the role of eosinophils in the pathogenesis of CSU. Kay et al. reported that CSU patients exhibit significantly increased numbers of eosinophils in non lesional skin as compared with control subjects, and IL-5 is increased in CSU skin lesions [11, 12]. Tissue factor expressed by eosinophils can induce the activation of blood coagulation generating thrombin which in turn can increase vascular permeability both directly, acting on endothelial cells, and indirectly, possibly by inducing degranulation of mast cells with release of histamine [22].

IL-5 induces the maturation, activation, and recruitment of eosinophils. Biologicals interfering with IL-5 and its receptor comprise benralizumab, an anti-IL-5Ra mAb, as well as mepolizumab and reslizumab, two anti-IL-5 mAbs. Unlike mepolizumab and reslizumab, benralizumab targets IL-5Ra and might affect leukocytes expressing low levels of IL-5Ra via antibody-dependent cell-mediated cytotoxicity [44]. A randomized, placebo-controlled parallel-group study was performed in 40 adult patients with moderate-to-severe atopic dermatitis, to evaluate the effect of a short term therapy with an anti-IL-5 antibody (mepolizumab; 2 × 750 mg). Unfortunately, despite a significant decrease in peripheral blood eosinophils, two single doses of 750 mg mepolizumab did not result in clinical success in atopic dermatitis patients [80]. There is an ongoing randomized, placebo-controlled, double-blind study (NCT01705795) evaluating the effect of anti-IL-5-therapy in patients with bullous pemphigoid. These anti-IL-5 biologicals should be assessed in future studies on CSU.

### Other targets

Targeting surface inhibitory receptors on mast cells might be a rational approach in treating allergic disorders. There are promising new candidate receptors as targets for treatment of mast cell-basophil mediated diseases. Among the inhibitory receptors, CD300a, FcγRIIB, and Siglec-8 have been shown to be expressed on MCs/Bs with promising preclinical results [81]. Selective targeting of CD300a receptor has been shown to be feasible for the treatment of mast cell and basophil mediated diseases [82]. The generation of agents targeting these receptors might also provide new insights in the treatment of CSU.

H4 receptors have been shown to modulate the function of mast cells and basophils, and in experimental models they show some promise in alleviating histamine-evoked itch [83–85]. A recent study explored the effectiveness of the combination of a H1R antagonist and H4R antagonist on chronic allergic dermatitis established in NC/Nga mice [86]. The combination of H1R antagonist olopatadine and H4R antagonist JNJ7777120 improved scratching behavior and was more effective than each of the antagonists individually. The effect of antihistamines on itch in the study was attributed to both the effect on inflammation (the treatment reduced the tissue mast cells, cytokines, and chemokines) and the direct effect on the itch-mediating pathways. The H4 receptor antagonists may be potential targets in treating urticaria as well as other allergic skin disorders.

Thymic stromal lymphopoietin (TSLP) is a TH2-initiating cytokine that activates mast cells by innate immune mechanisms. TSLP has been shown to be increased in lesional but not nonlesional skin of CSU patients [11]. AMG 157 is a human monoclonal antibody that blocks the interaction of TSLP with its receptor and has been investigated in patients with atopic dermatitis in a placebo-controlled, randomized, double blind study (NCT00757042). The study has been completed but the results have not been published. Drugs that target the TSLP–TSLPR signaling axis via pharmacological inhibition or by antibody-mediated neutralization of TSLP, could also be an option to treat CSU patients.

Cellular adhesion molecules (CAMs) have been implicated as potential sustainers of the late phase of CSU by selective recruitment and activation of inflammatory cells leading to tissue damage. ICAM-1, ELAM-1 and VCAM-1 showed an upregulation in CSU and P-selectin levels were elevated in both CSU and dermographism, but the most relevant finding at the cutaneous level seemed to be the strong production of CAMs also in unaffected skin. The elevation of CAMs in the weals as well as in unaffected skin has been interpreted as a sign of minimally persistent inflammation in patients with urticaria [87–90]. Cell adhesion inhibitors such as natalizumab (monoclonal antibody against α-4-integrin) may have a role in the treatment of CSU in the future.

IgE synthesis is suppressed by inhibition of the cytokines IL-4 or IL-13, therefore there have been attempts to influence IgE production at these steps. Biologicals directed against IL-4Rα receptors are AMG-317, dupilumab and pitrakinra [44]. IL-13 targeting biologicals encompass several anti-IL-13 mAbs including ABT-308, anrukinzumab, IMA-026, lebrikizumab, CNTO, 5825, GSK679586, QAX576 and tralokinumab [44]. The elevated levels of IL-4 and IL-13 in CSU patients have been reported before [91–93]. Therefore the agents targeting IL-4 and IL-13, such as dupilumab, might have a role in the treatment of CSU in the future.

**Fig. 1:**
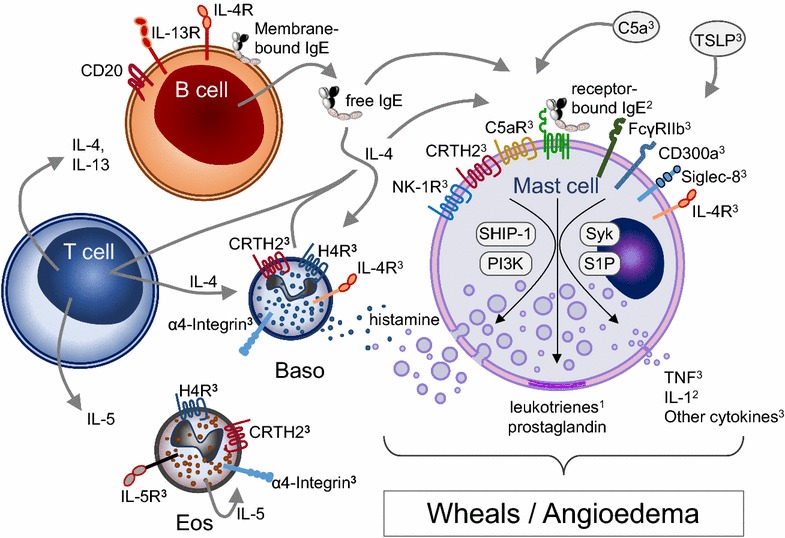
Potential targets in the treatment of chronic urticaria. *Baso* basophil, *CRTH* chemoattractant receptor-homologous molecule expressed on Th2 cells (DP2), *Eos* eosinophil, *H1/4R* histamine 1/4 receptor, *NK* neurokinin, *C5* complement 5, *IgE* immunoglobulin, *IL* interleukin, *LTR* leukotriene receptor, *PI3* *K* phosphoinositide 3-kinase, *S1P* sphingosine-1-phosphate, *SHIP* SH2-containing inositol phosphatase 1, *Syk* spleen tyrosine kinase, *TSLP* thymic stromal lymphopoietin. ^1^Currently available, ^2^under investigation, ^3^hypothetical

Possible future targets for treatment are given in Table 2. Figure 1 shows potential targets in the treatment of CSU.

**Table 2:** Possible future targets for the treatment of CSU

Target	Drug	Proposed mechanism of action
NK-1R	Aprepitant, tradipitant, serlopitant, orvepitant	Small molecules which bind to neurokinin-1 receptors and thus block substance P activity
C5a	Eculizumab	Reduces mediator release in autoimmune urticaria
H4 receptor	JNJ7777120	Reduces histamine mediated itch
TNF-α	Etanercept, infliximab, adalimumab	Reduces chemotaxis, decrease inflammation, decrease angiogenesis
TSLP	Tezepelumab	Inhibits release of Th2-cytokines
α_4_-integrin	Natalizumab	Inhibits endotelial activation
α_4_β_7_-integrin	Vedolizumab	Inhibits endotelial activation
β7 integrin	RhuMab β7	Inhibits endotelial activation
CD-20	Rituximab, ofatumumab, ocrelizumab	Depletes antibody-producing B cells
IL-4Rα	Dupilumab, pitrakinra, AMG-317	Reduces IgE production
IL-13	ABT-308, anrukinzumab, IMA-026, lebrikizumab, CNTO,5825, GSK679586, QAX576, tralokinumab	Reduces IgE production
IL-5Rα	Benralizumab	Inhibits eosinophil activation
IL-5	Mepolizumab, reslizumab	Inhibits eosinophil activation

## Conclusion

CSU is a chronic disabling inflammatory skin disease, which is in many cases well-controlled by the existing licensed treatment options. In approximately 1 of 5 CSU patients, these treatment options are not sufficient. Novel drugs are needed and are under development. Ligelizumab, PGD2 receptor antagonists, a topical Syk inhibitor, and canakinumab are promising candidates for future CSU treatment options and are currently being tested in clinical trials for their efficacy and safety in CSU. Substance P antagonists, DARPins, blockers of C5a/C5aR, therapies targeting IL-4, IL-5 and IL-13, and drugs that target inhibitory mast cell receptors should be tested in controlled CSU trials. Many other mediators and receptors are held to be of pathogenic relevance, and this should be explored in skin profiling studies and functional proof of concept studies.

One important point must not be forgotten when we search for new and better medication for the prevention and symptomatic treatment of CSU; the ultimate goal is to develop strategies and drugs that can cure CSU, rather than stop the signs and symptoms. The exploration of novel therapeutic targets for their role and relevance in CSU can help to achieve this, by providing a better understanding of its etiopathogenesis.

## Abbreviations

CSU: chronic spontaneous urticaria
IL-1: interleukin-1
TNF: tumor necrosis factor
DARPins: designed ankyrin repeat proteins
SHIP: Src homology inositol phosphatase
Syk: spleen tyrosine kinase
LTRA: leukotriene antagonists
CRTH2: chemoattractant receptor homologous molecule expressed on Th2 cells
DP: D-type prostanoid receptor
PGD2: prostaglandin D2
SP: substance P
NKR: neurokinin receptors
TSLP: thymic stromal lymphopoietin
CAMs: cellular adhesion molecules
