# Erratum to: Looking forward to new targeted treatments for chronic spontaneous urticaria

**DOI:** 10.1186/s13601-017-0148-9

**Published:** 2017-04-03

**Authors:** Emek Kocatürk, Marcus Maurer, Martin Metz, Clive Grattan

**Affiliations:** 1 Department of Dermatology, Okmeydanı Training and Research Hospital, Istanbul, Turkey; 2 Department of Dermatology and Allergy, Charité – Universitäts medizin, Berlin, Germany; 3 St John’s Institute of Dermatology, Guy’s Hospital, London, UK

## Erratum to: Clin Transl Allergy (2017) 7:1 DOI 10.1186/s13601-016-0139-2

Information from the manufacturer of the topical intervention in the GSK2646264 trial outlined in this review [1] indicates that the half-life of the product has not been established with certainty on the available data.
